# Supplementary material for: Stability of the personal relationship networks in a longitudinal study of middle school students
Source: Sci Rep. 2023 Sep 4;13:14575. doi: 10.1038/s41598-023-41787-x (PMC10477262; doi:10.1038/s41598-023-41787-x)
Supplement: Supplementary file 1 — Supplementary Figures. [file 41598_2023_41787_MOESM1_ESM.pdf]

Supplementary Information on "Stability of the personal relationship networks in a longitudinal study of middle school students"

Diego Escribano,[1] Francisco J. Lapuente,[2,3] José A. Cuesta,[1,4], Robin I.M. Dunbar[5] and Angel Sánchez[1,4]

[1]Grupo Interdisciplinar de Sistemas Complejos (GISC), Departamento de Matemáticas, Universidad Carlos III de Madrid, 28911 Leganés, Madrid, Spain

[2]Instituto de Enseñanza Secundaria Blas de Otero, 28024 Madrid, Spain

[3]Departamento de Biología y Geología, Física y Química Inorgánica, Universidad Rey Juan Carlos, 28933, Móstoles, Madrid, Spain

[4]Instituto de Biocomputación y Física de Sistemas Complejos (BIFI), Universidad de Zaragoza, 50018 Zaragoza, Spain

[5]Department of Experimental Psychology, University of Oxford, Oxford OX2 6GG, UK

S1 Number of responders across groups

In our research, we have students responding to our surveys during the academic years 2020-2021 and 2021-2022. To identify them uniquely, we associate each student with the course they are currently taking in the year 2021-2022. As we are only considering students that answered all five surveys, this implies that there are practically no students in 1st-year ESO, because they only participated in waves 4 and 5. Those that are still listed as being in 1st-year ESO are therefore "repetidores".

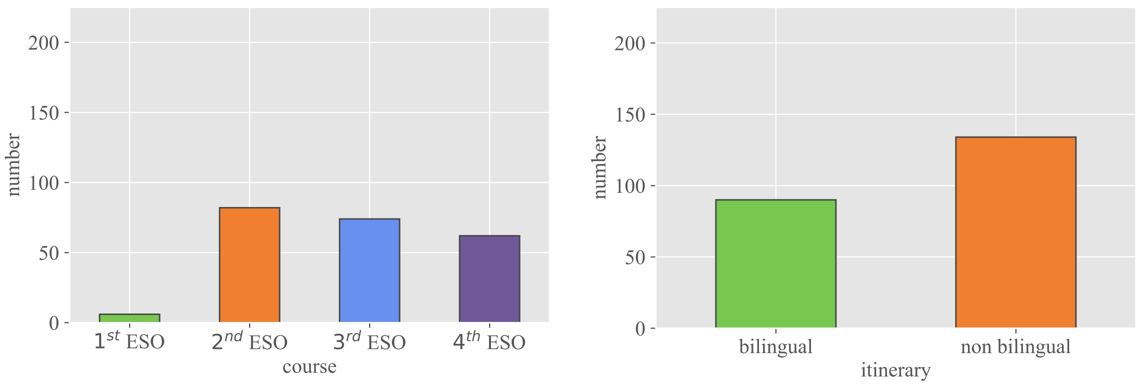

Fig. S1: **Distribution of the students participating in all five waves -** Left: by course. Right: by itinerary.

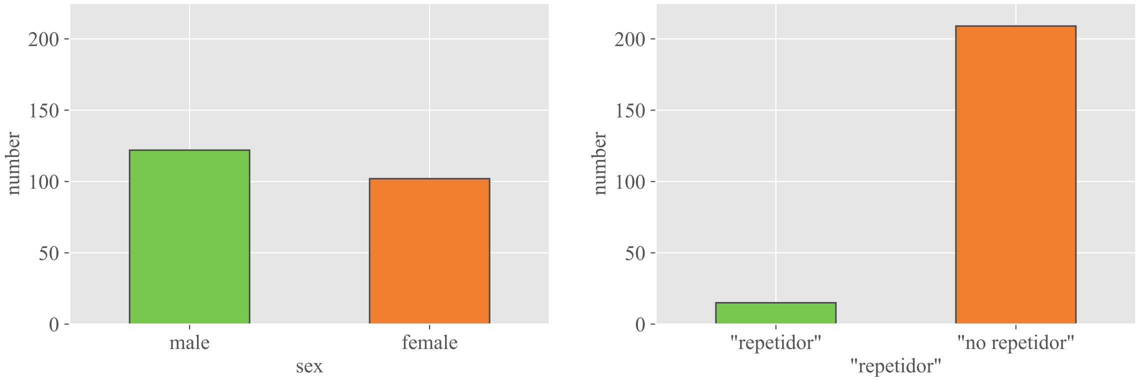

Fig. S2: **Distribution of the students participating in all five waves -** Left: by sex. Right: by “repetidores” or not.

S2 Number of friendships

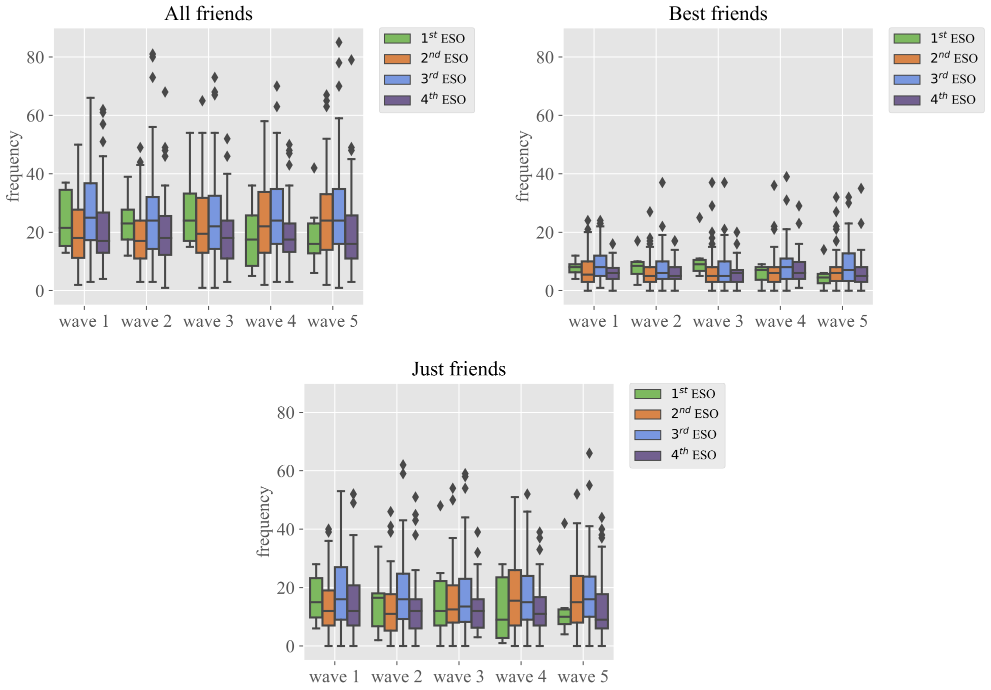

Fig. S3: **Number of friendships declared by course -** Left: all friends. Right: best friends. Bottom: just friends.

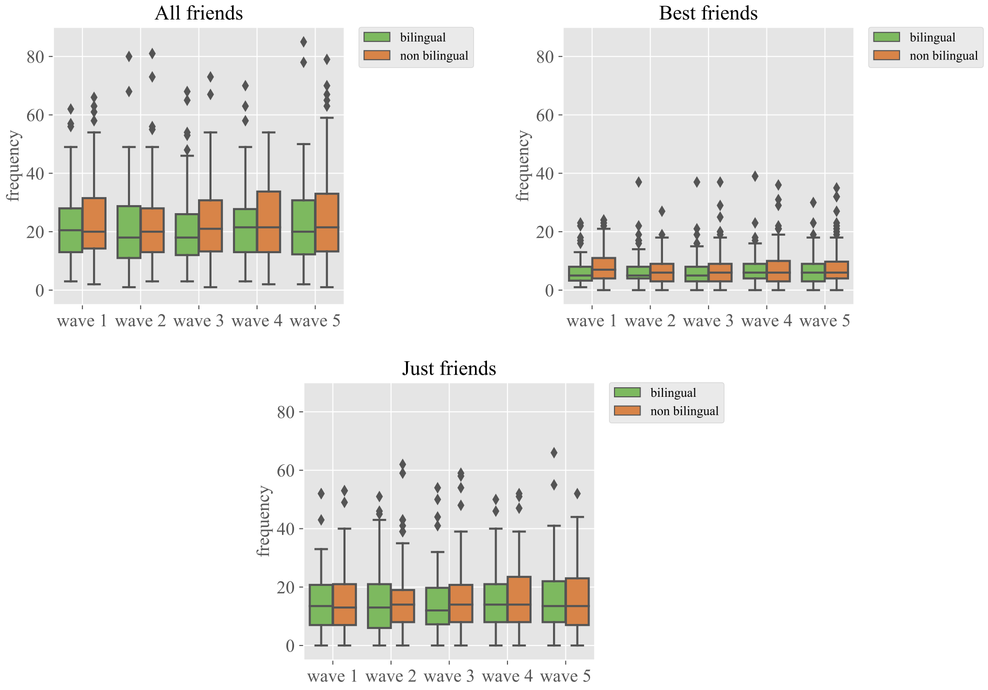

Fig. S4: **Number of friendships declared by itinerary** - Left: all friends. Right: best friends. Bottom: just friends.

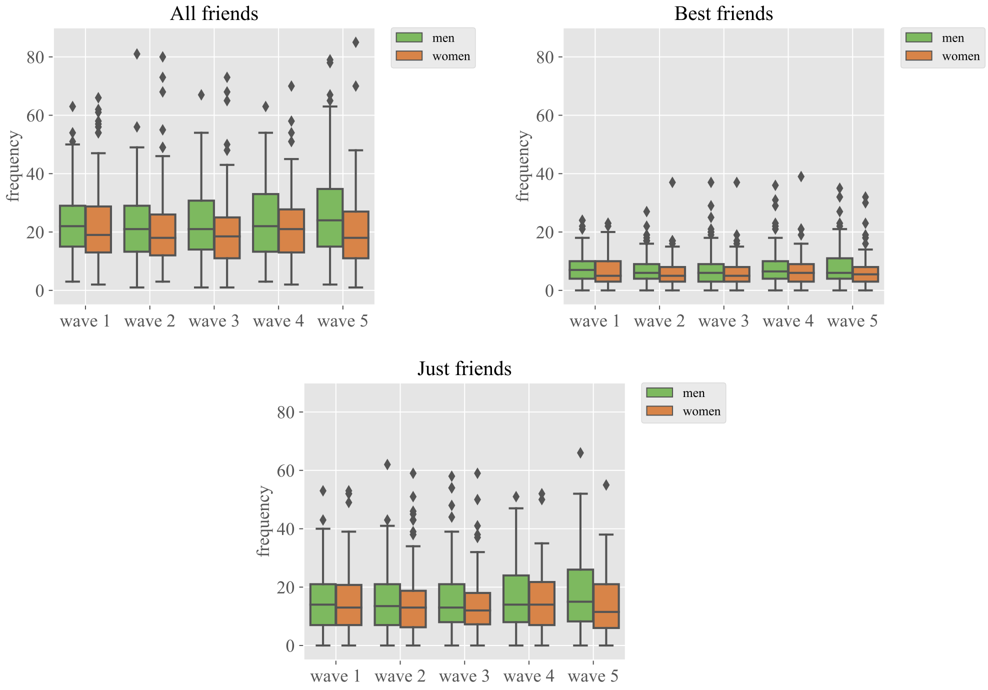

Fig. S5: **Number of friendships declared by gender** - Left: all friends. Right: best friends. Bottom: just friends.

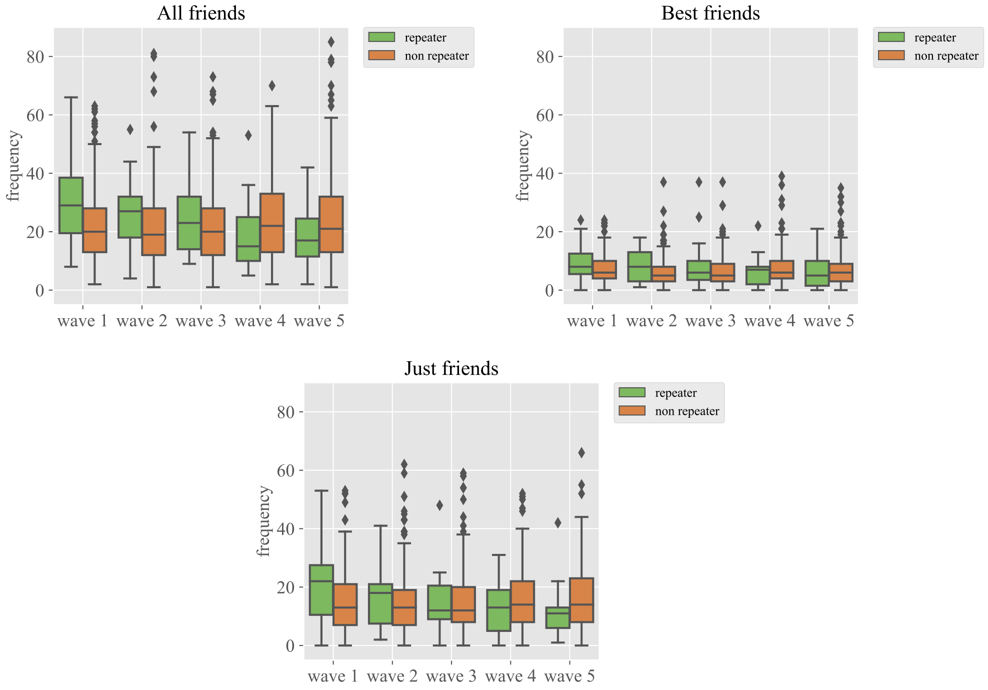

Fig. S6: **Number of friendship declared by "repetidores" or not -** Left: all friends. Right: best friends. Bottom: just friends.

S3 μ parameter.

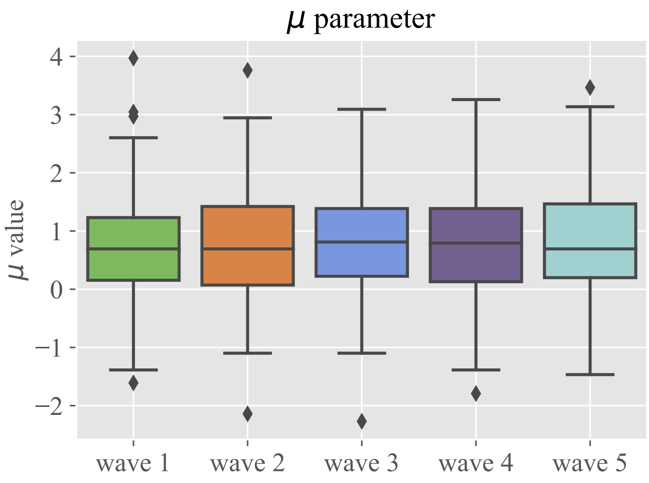

Fig. S7: μ **parameter distribution across waves**

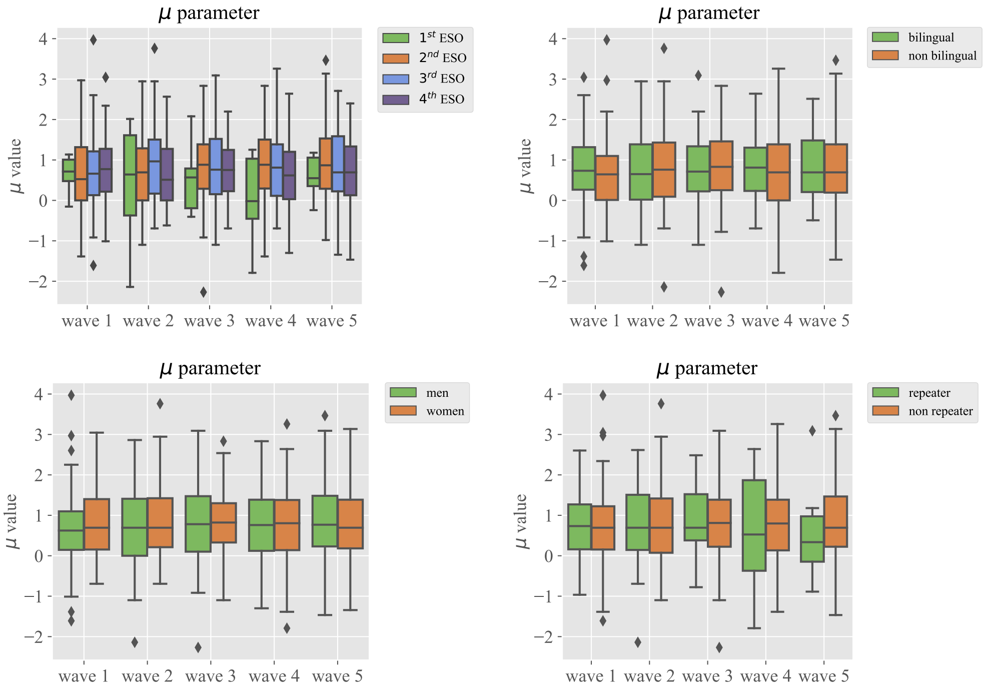

Fig. S8: μ **parameter distribution with division by groups** - Top left: by course. Top right: by itinerary. Bottom left: by gender. Bottom right: by "repetidores" or not.

S4 Slopes of the linear fit to aggregate evolution over time

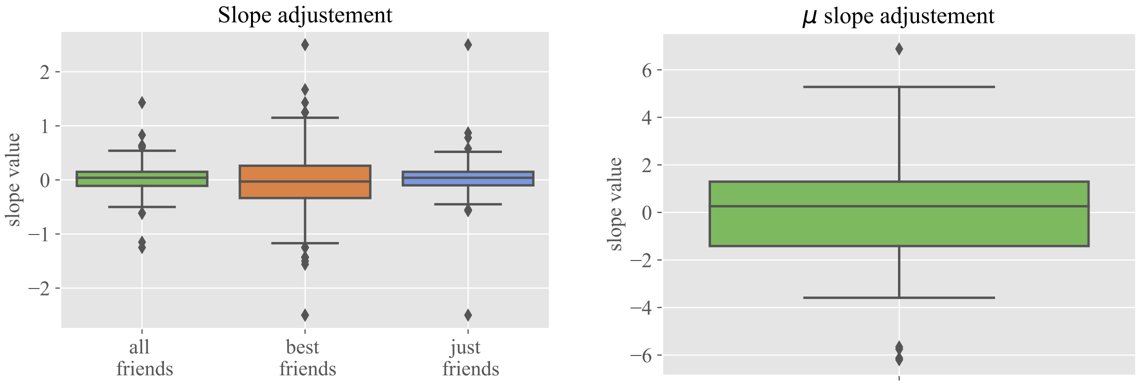

Fig. S9: **Slope of linear fits to the evolution** - Left: number of friendships. Right: μ parameter.

S5 Transitions of relationships between waves

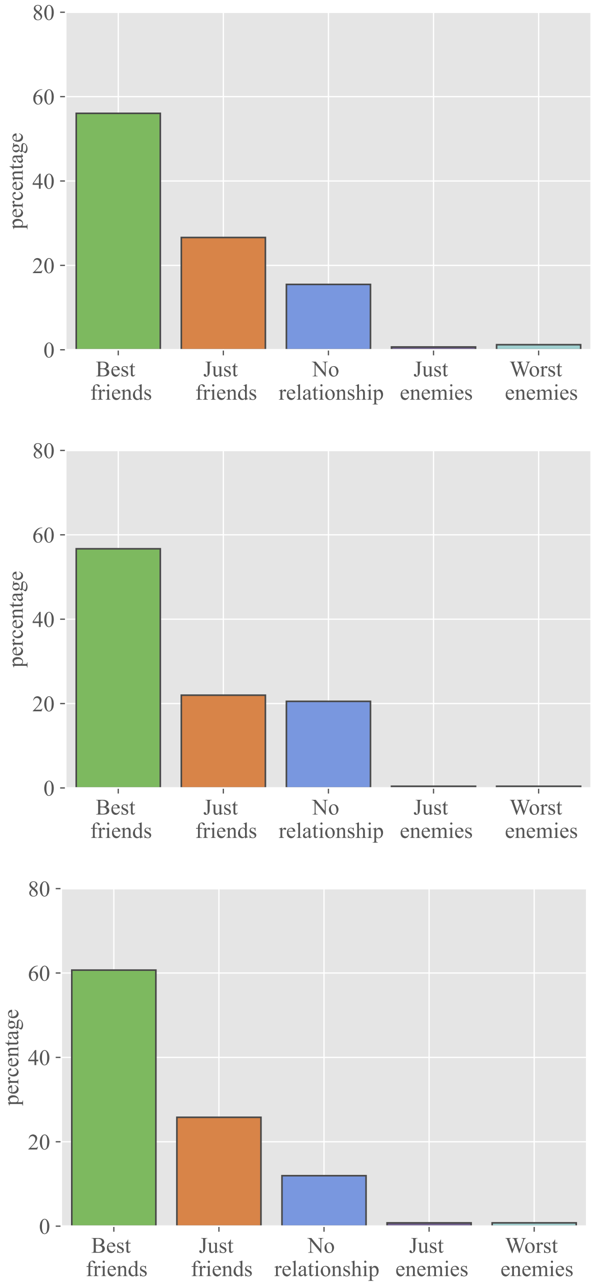

Fig. S10: **Best friends transitions -** Percentage of individuals that ended up in a given category in wave n, when they were marked as "best friend" in the previous wave (conditional probability $P(x, w_n| + 2, w_{n-1})$). Top: from wave 1 to wave 2. Middle: from wave 2 to wave 3. Bottom: from wave 3 to wave 4.

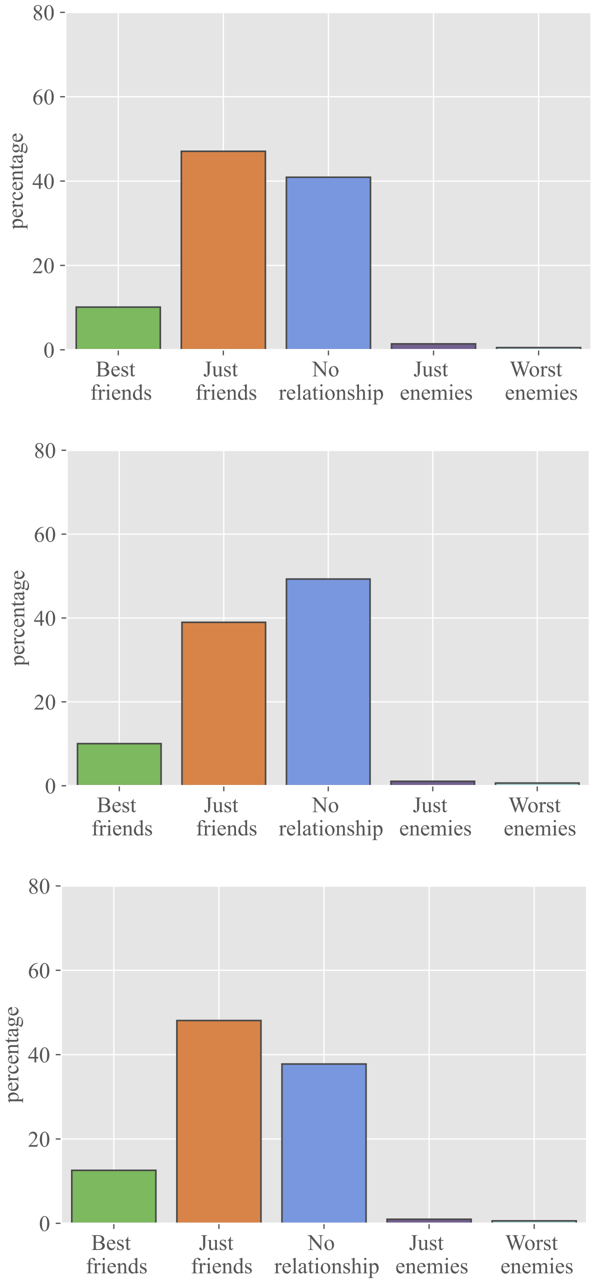

Fig. S11: **Friends transitions** - Percentage of individuals that ended up in a given category in wave n, when they were marked as "just friend" in the previous wave (conditional probability $P(x, w_n| + 1, w_{n-1})$). Top: from wave 1 to wave 2. Middle: from wave 2 to wave 3. Bottom: from wave 3 to wave 4.

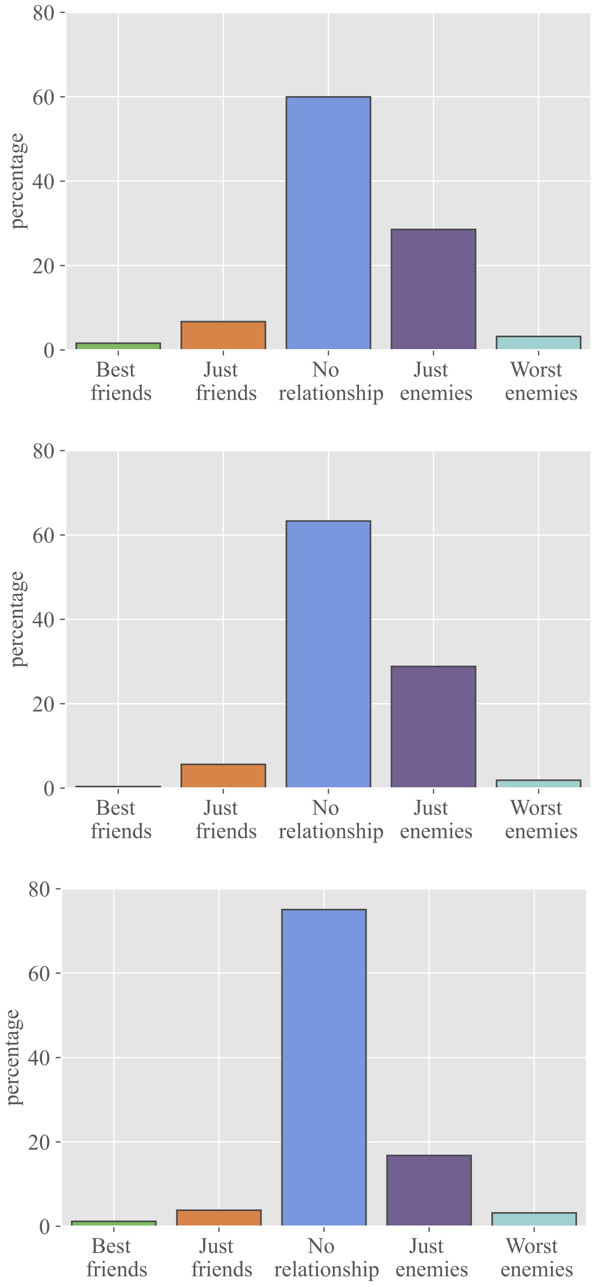

Fig. S12: **Enemies transitions -** Percentage of individuals that ended up in a given category in wave n, when they were marked as “just enemy” in the previous wave (conditional probability $P(x, w_n| -1, w_{n-1})$). Top: from wave 1 to wave 2. Middle: from wave 2 to wave 3. Bottom: from wave 3 to wave 4.

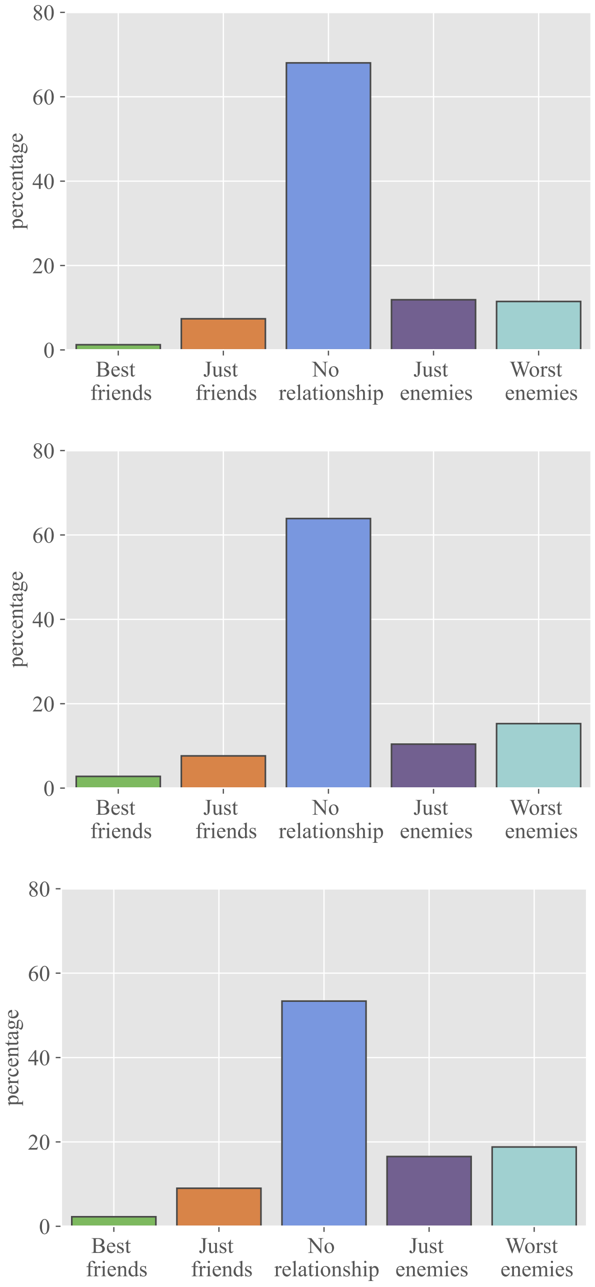

Fig. S13: **Worst enemies transitions -** Percentage of individuals that ended up in a given category in wave n, when they were marked as "worst enemy" in the previous wave (conditional probability $P(x, w_n| - 2, w_{n-1})$). Top: from wave 1 to wave 2. Middle: from wave 2 to wave 3. Bottom: from wave 3 to wave 4.

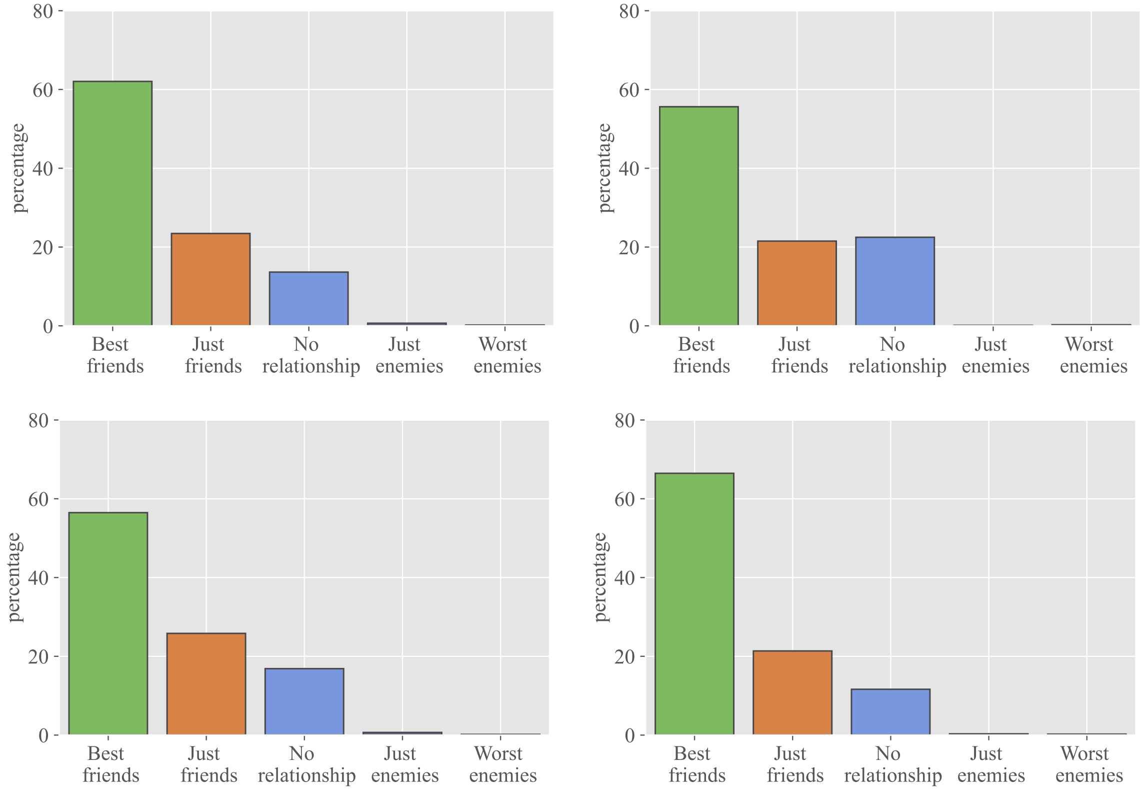

Fig. S14: **Best friends origin** - Percentage of individuals that end up as "best friends" in wave n and were marked in any category in the previous wave (conditional probability $P(x, w_{n-1}|2, w_n)$). Top left: from wave 1 to wave 2. Top right: from wave 2 to wave 3. Bottom left: from wave 3 to wave 4. Bottom right: from wave 4 to wave 5.

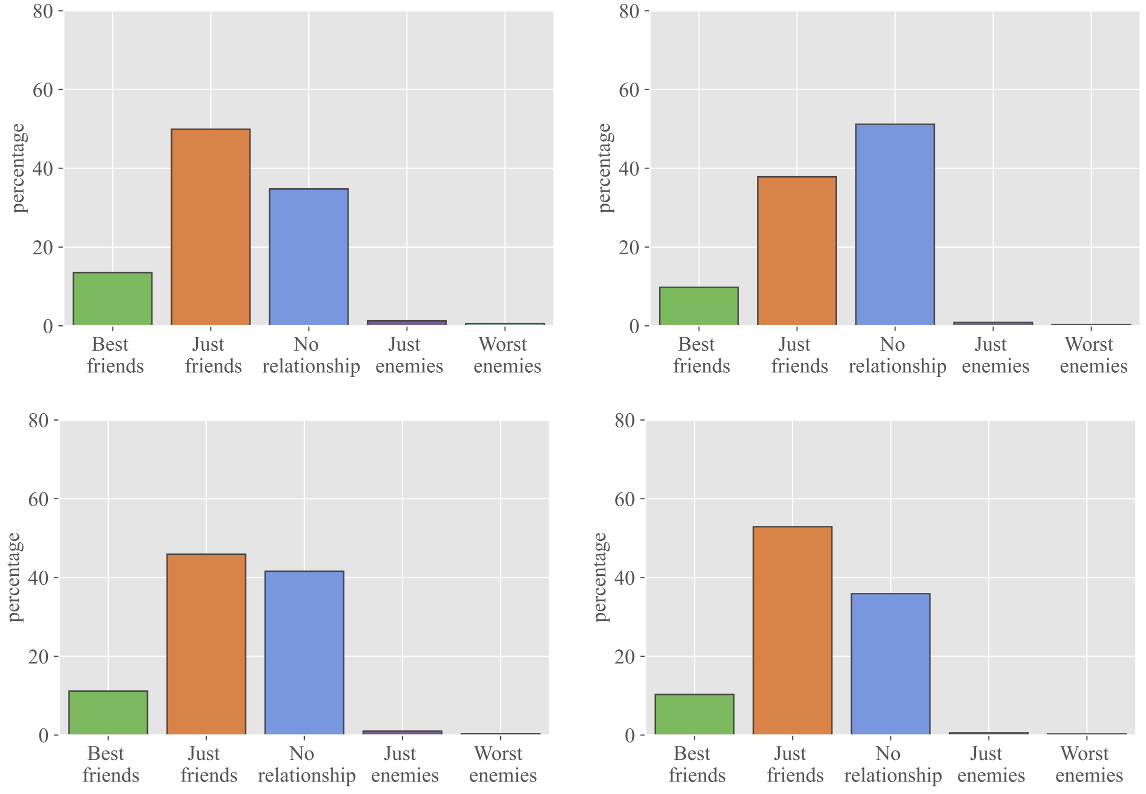

Fig. S15: **Just friends origin -** Percentage of individuals that end up as "just friends" in wave n and were marked in any category in the previous wave (conditional probability $P(x, w_{n-1}|1, w_n)$). Top left: from wave 1 to wave 2. Top right: from wave 2 to wave 3. Bottom left: from wave 3 to wave 4. Bottom right: from wave 4 to wave 5.

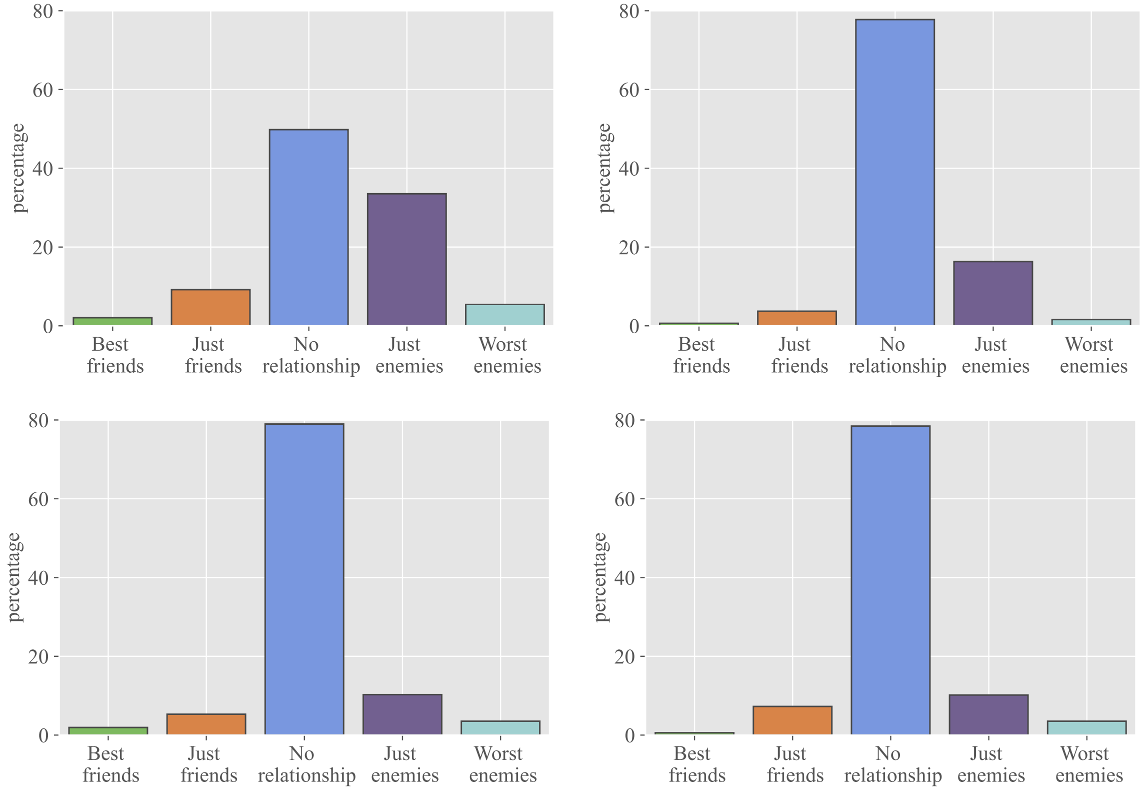

Fig. S16: **Just enemies origin -** Percentage of individuals that end up as "just enemies" in wave n and were marked in any category in the previous wave (conditional probability $P(x, w_{n-1}| -1, w_n)$). Top left: from wave 1 to wave 2. Top right: from wave 2 to wave 3. Bottom left: from wave 3 to wave 4. Bottom right: from wave 4 to wave 5.

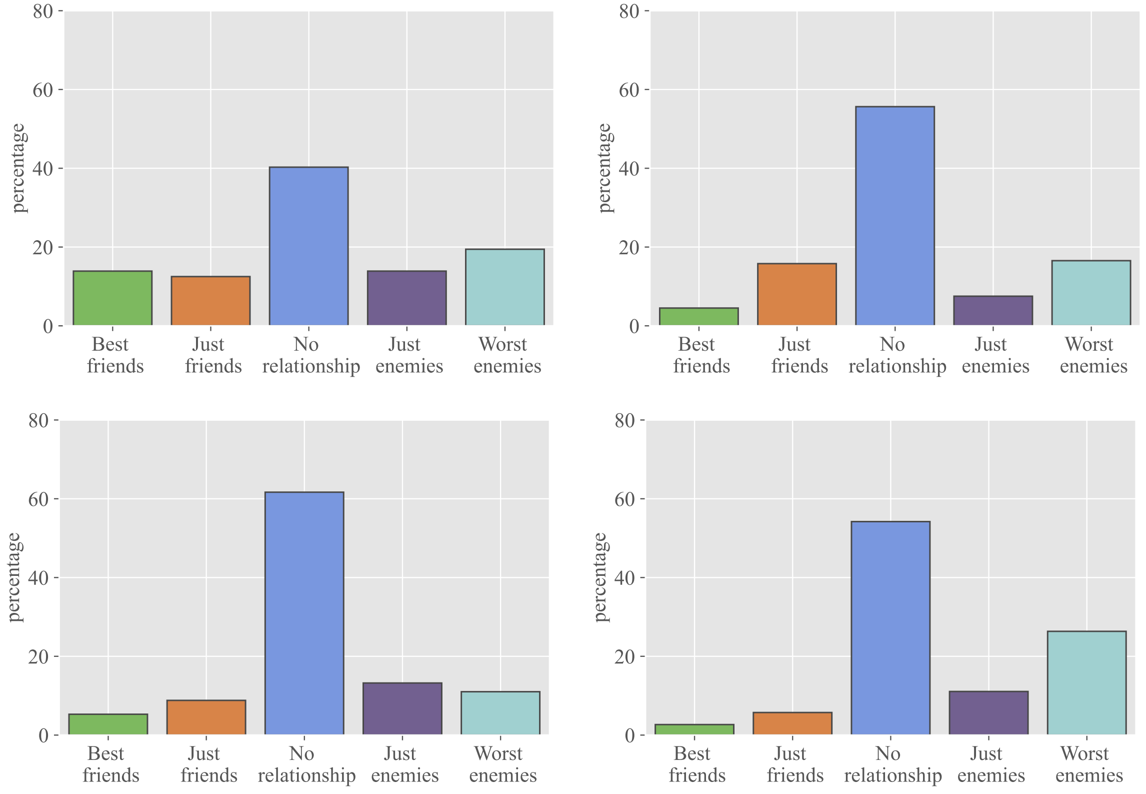

Fig. S17: **Worst enemies origin -** Percentage of individuals that end up as "worst enemies" in wave n and were marked in any category in the previous wave (conditional probability $P(x, w_{n-1}|-2, w_n)$). Top left: from wave 1 to wave 2. Top right: from wave 2 to wave 3. Bottom left: from wave 3 to wave 4. Bottom right: from wave 4 to wave 5.

S6 Number of S-S, S-D, D-S, and D-D relationships

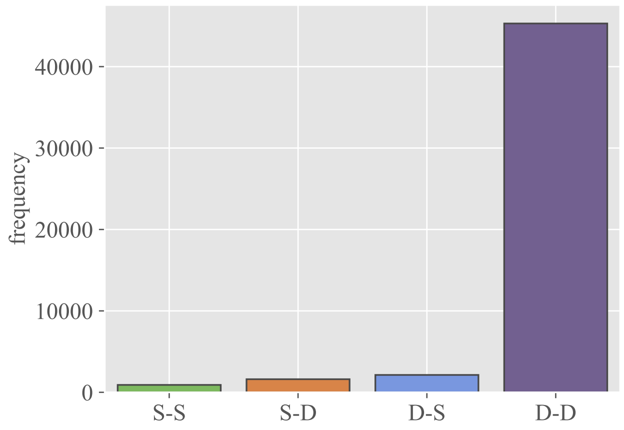

Fig. S18: **Number of pairs of relationships -** S-S: same class in both academic years. S-D: same class the first year and different the second. D-S: different class the first year and same the second. D-D: different classes in both academic years.

S7 Reciprocity

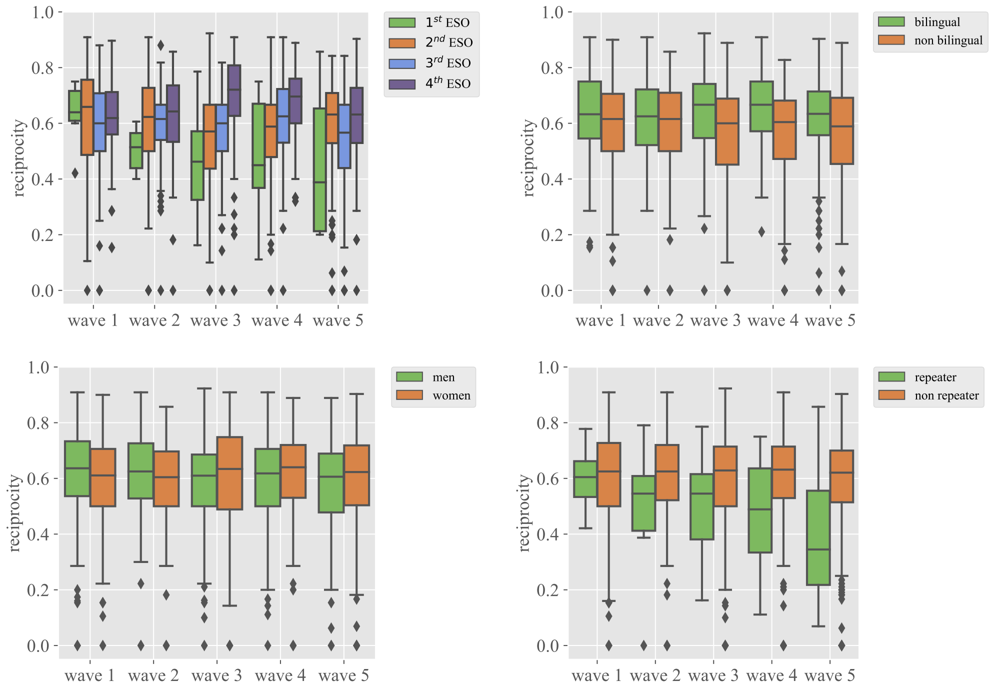

Fig. S19: **Reciprocal relationships -** Top left: by course. Top right: by itinerary. Bottom left: by gender. Bottom right: by "repetidores" or not.

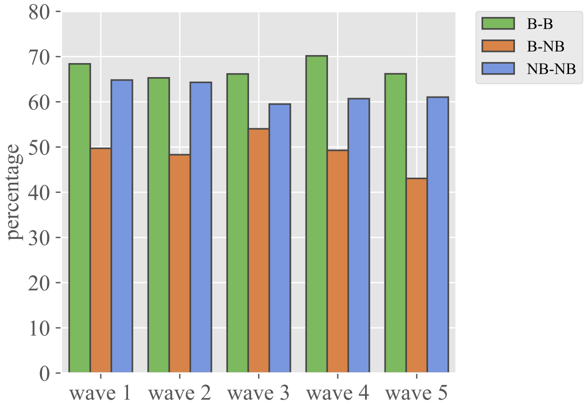

Fig. S20: **Reciprocity per itinerary** - B-B (bilingüe-bilingüe), B-NB (bilingüe-no bilingüe), NB-NB (no bilingüe-no bilingüe).

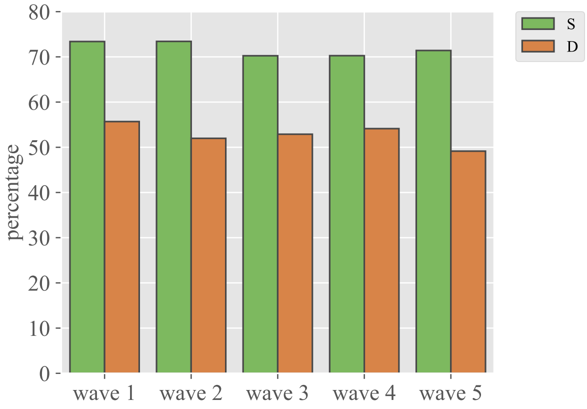

Fig. S21: **Reciprocity per group** - S (same group), D (different group).

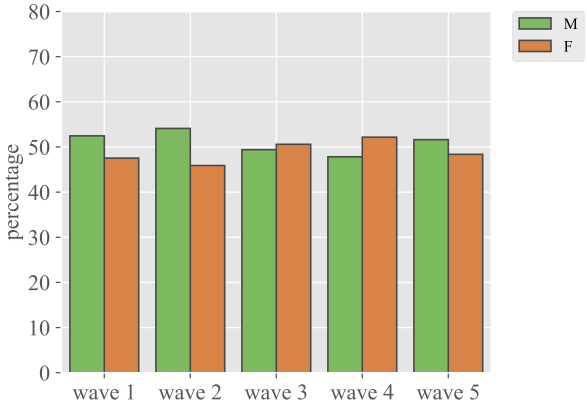

Fig. S22: **Reciprocity per sex** - Percentage of non-reciprocal relationships that are directed from men to women (green) and from women to men (orange) in each wave.

S8 Reciprocity of negative relationships

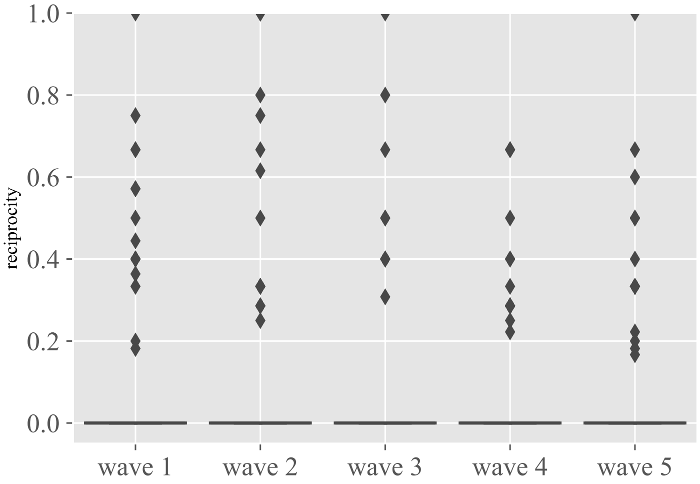

Fig. S23: **The structure of friendships** - Replica of Figure 1 in the main manuscript without filtering outliers.

S9 Analysis without removing outliers

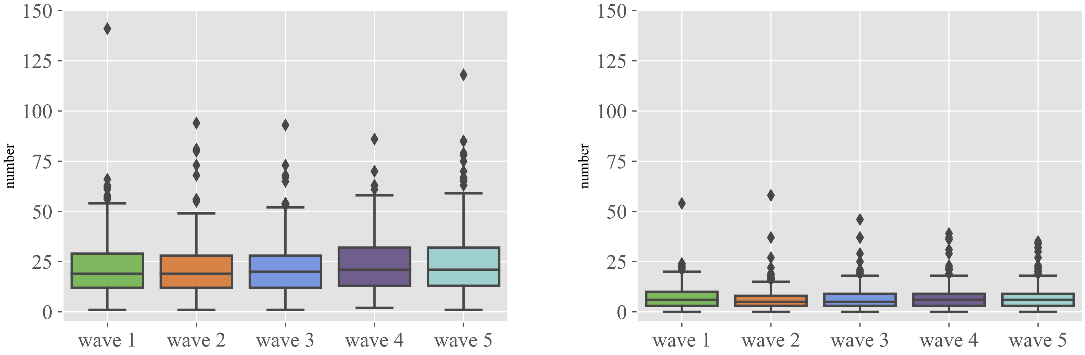

Fig. S24: **The structure of friendships** - Replica of Figure 1 in the main manuscript without filtering outliers.

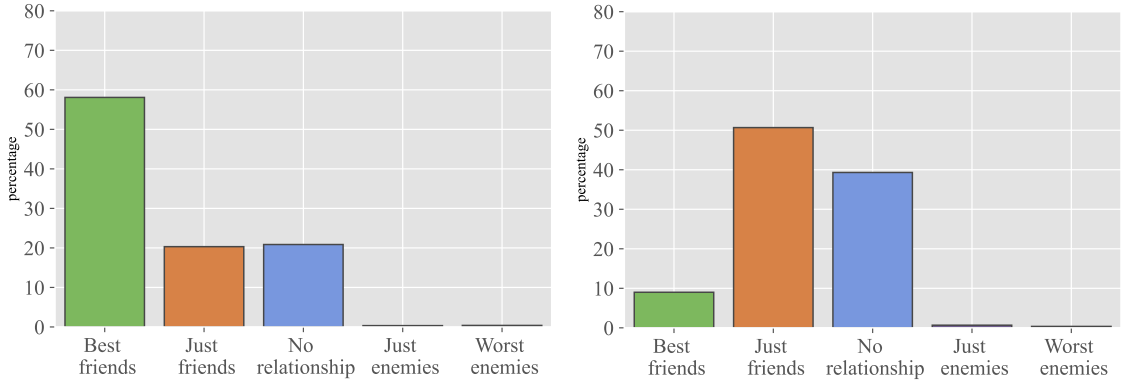

Fig. S25: **The evolution of friendships over time** - Replica of Figure 2 in the main manuscript without filtering outliers.

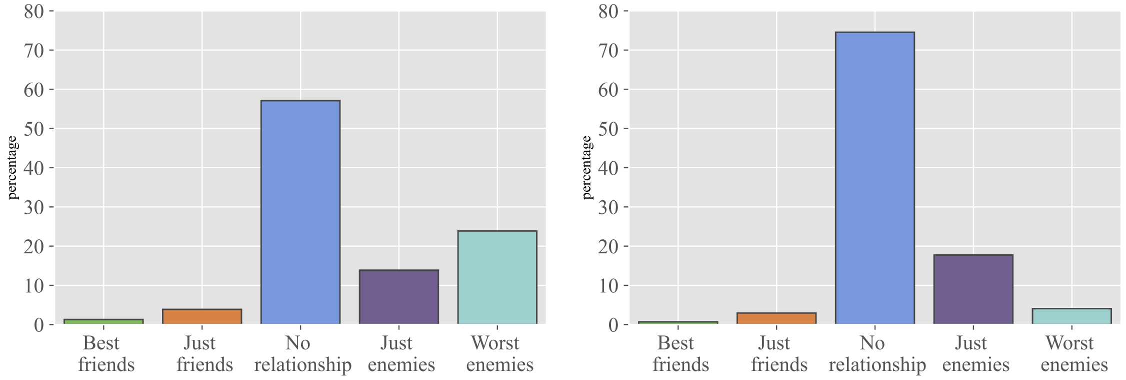

Fig. S26: **The evolution of enmities over time** - Replica of Figure 3 in the main manuscript without filtering outliers.

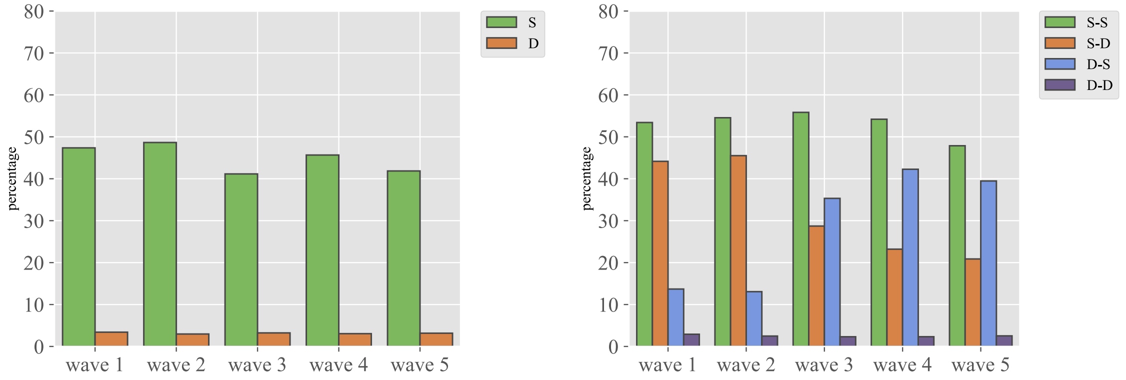

Fig. S27: **The importance of the group for the existence of relationships** - Replica of Figure 4 in the main manuscript without filtering outliers.

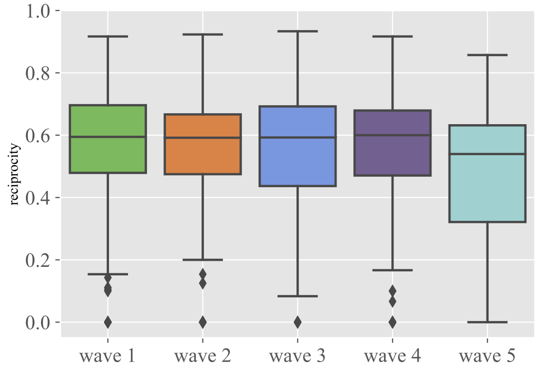

Fig. S28: **The evolution of reciprocity over time -** Replica of Figure 5 in the main manuscript without filtering outliers.

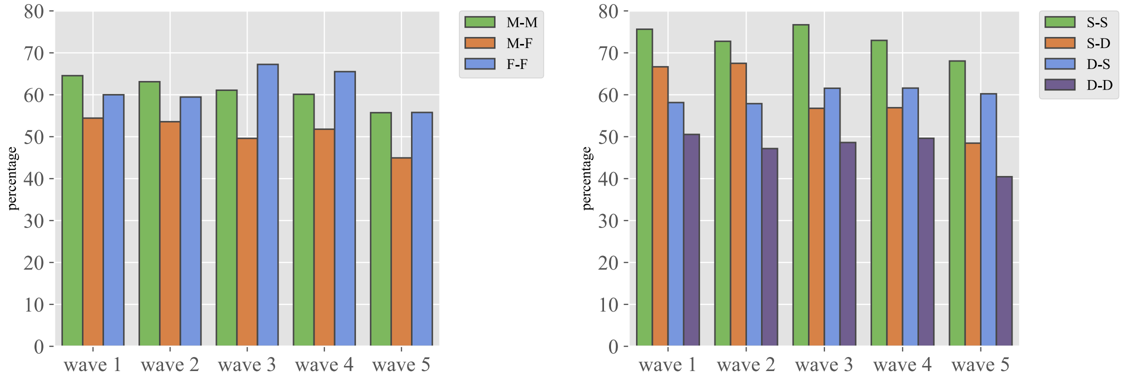

Fig. S29: **The importance of the group for the reciprocity of relationships -** Replica of Figure 6 in the main manuscript without filtering outliers.
